# Ileal Amino Acid Digestibility in Various Protein Sources Fed to Broiler Chickens

**DOI:** 10.3390/ani16050779

**Published:** 2026-03-02

**Authors:** Inho Cho, June Hyeok Yoon, Hyun Jung Jung, Changsu Kong

**Affiliations:** 1 Department of Animal Science and Biotechnology, Kyungpook National University, Sangju 37224, Republic of Korea; inhoblog970630@gmail.com; 2 Department of Poultry Science, University of Georgia, Athens, GA 30602, USA; junehyeokyoon@gmail.com; 3 Precision Animal Nutrition Division, National Institute of Animal Science, Rural Development Administration, Wanju 55365, Republic of Korea; hyjjung@korea.kr; 4 Department of Animal Science, Kyungpook National University, Sangju 37224, Republic of Korea; 5 Research Institute for Innovative Animal Science, Kyungpook National University, Sangju 37224, Republic of Korea

**Keywords:** amino acid, apparent ileal digestibility, broiler, protein source, standardized ileal digestibility

## Abstract

This study aimed to determine the ileal digestibility of amino acids (AA) in various protein sources for 21-day-old broilers. A total of 448 Ross 308 male broilers were allocated to eight dietary treatments with eight replicates in a randomized complete block design. Experimental diets included one nitrogen-free diet and seven test diets, each containing one of the following feed ingredients—dehulled soybean meal (SBM), fermented SBM (FSBM), rapeseed meal (RM), copra meal (CM), palm kernel meal (PKM), corn distillers dried grains with solubles (DDGS), and fish meal (FM), as the sole source of AA. On day 21, all birds were euthanized and subsequently ileal digesta was collected from the distal two-thirds of the ileum, extending from Meckel’s diverticulum to 1 cm proximal to the ileocecal junction. The ileal digestibility of AA in the FM was the greatest, followed by the SBM. The ileal digestibility for AA in the SBM was greater than that in the RM. The ileal AA digestibility in the RM was greater than or not different from that in the FSBM, except for Val and Pro, and superior to the CM and the PKM. The ileal digestibility of AA in the FSBM was greater than or not different from those in corn DDGS, except for Met and Cys. Corn DDGS exhibited greater or not different ileal digestibility of AA compared to that of the CM and the PKM, except for Val and Asp, and the PKM was the lowest. In conclusion, the ileal digestibility of AA was the greatest in the FM, followed by the SBM, FSBM, the RM, corn DDGS, the CM, and the PKM. Furthermore, the results underscore the necessity for continuous evaluation of ileal AA digestibility in various protein sources.

## Simple Summary

This study determines the ileal digestibility of amino acids (AA) in seven protein sources, including dehulled soybean meal (SBM), fermented SBM (FSBM), rapeseed meal (RM), copra meal (CM), palm kernel meal (PKM), corn distillers dried grains with solubles (DDGS), and fish meal (FM), in 21-day-old broilers. The results show that the FM has the greatest ileal AA digestibility among all protein sources, followed by the SBM, the RM, the FSBM, corn DDGS, the CM, and the PKM. The range of standardized ileal digestibility of AA for each source was 77.2% (Cys) to 98.7% (Lys) for the FM, 75.7% (Cys) to 89.4% (Arg) for the SBM, 69.5% (Pro) to 84.3% (Met) for the RM, 54.5% (Cys) to 83.3% (Lys) for the FSBM, 59.9% (Asp) to 85.4% (Met) for corn DDGS, 53.6% (Cys) to 80.3% (Val) for the CM, and 40.9% (His) to 82.1% (Met) for the PKM. These findings highlight the necessity of accurate evaluation of the ileal AA digestibility in various protein sources for optimal broiler diet formulation.

## 1. Introduction

In poultry diet, the main protein source to satisfy amino acids (AA) requirements is soybean meal (SBM). However, the cost of the SBM is projected to increase due to climate change [1] and increased demand resulting from elevated meat production [2,3,4]. Consequently, corn distillers dried grains with solubles (DDGS) emerged as a more cost-effective alternative to the SBM in the mid-2000s, given its lower cost and the presence of valuable nutritive components such as protein, fat, fiber, and minerals [5]. This diversification of protein sources not only offers a pathway to reduce feed costs but also contributes to the establishment of a more sustainable livestock system [6,7]. In addition to corn DDGS, alternative protein sources such as the palm kernel meal (PKM), the rapeseed meal (RM), and the copra meal (CM) have also been proposed as substitutes for the SBM in poultry diets.

Despite the benefits of using various alternative protein sources, practical implementation has been challenging. Accurately evaluating AA composition and AA utilization of feed ingredients is crucial to sufficiently provide AA required by animals; however, such feed ingredient evaluation of alternative protein sources has not been sufficiently conducted, which limits their practical application in broiler diet formulation. To accurately assess AA utilization, determining digestible AA content in protein sources is more appropriate than formulating diets based on total AA [8,9]. The digestibility of AA in feed ingredients for broilers and pigs is commonly assessed as ileal digestibility to avoid the effects of microbial AA fermentation in hindgut [10]. Ileal digestibility is measured as either apparent ileal digestibility (AID), which does not account for the basal endogenous losses (BEL) of AA, or as standardized ileal digestibility (SID), which corrects for these losses. Although major feed ingredients like corn and the SBM have been extensively evaluated, data on the AID and SID of AA in various alternative protein sources remain limited. Therefore, to bridge this gap, the aim of the present study was to determine the ileal digestibility of AA in various protein sources for 21-day-old broilers. The null hypothesis was that there would be no differences in apparent and standardized ileal digestibility of AA among the tested protein sources in 21-day-old broilers.

## 2. Materials and Methods

Experimental procedures were conducted in accordance with the Institutional Animal Care and Use Committee of Kyungpook National University, Republic of Korea (approval number: KNU 2023-0109).

### 2.1. Animals Experiment and Management

Post-hatch male Ross 308 broilers were assigned identification tags and housed in battery cages (60 cm × 50 cm × 60 cm) within an environmentally controlled facility that operated under a continuous 24 h lighting program. Birds were fed a crumbled pre-starter diet (220 g/kg CP and 12.6 MJ/kg AMEn) until day 7. Subsequently, the starter diet (200 g/kg CP and 13 MJ/kg AMEn) was provided until day 17. On day 17, a total of 448 birds (626 ± 64.4 g) were allocated to eight dietary treatment groups, with eight replicate cages per treatment (seven birds per cage), in a randomized complete block design. The birds were fed the experimental diets in mash form from day 17 to day 21, with ad libitum access to both feed and water. Room temperature was maintained at 33 °C for the first 3 days and then gradually decreased by 3 °C per week until it reached 24 °C. During the experimental period, relative humidity was maintained at approximately 50%, with fluctuations ranging from about 40% to 60%.

### 2.2. Ingredients and Dietary Treatments

Eight experimental diets consisted of one nitrogen-free diet (NFD) and seven test diets, each containing one of the following feed ingredients such as the dehulled SBM, fermented SBM (FSBM), the RM, the CM, the PKM, corn DDGS, and the fish meal (FM) (Table 1). Feed ingredients were obtained in mash form from a local supplier. The NFD was formulated to estimate the BEL of AA, as previously described by Adedokun et al. [11]. Seven dietary treatments were formulated, which contained a single protein source as the sole contributor of AA. The concentration of the test ingredients in experimental diets was adjusted to ensure a dietary CP level of a maximum of 20%, based on analyzed values, and experimental diets were formulated to meet mineral requirements using analyzed values. Chromium oxide was added at 5 g/kg to all diets as an index.

### 2.3. Sample Collection

On day 21, all birds were euthanized via CO_2_ asphyxiation. Ileal digesta samples were subsequently collected from the distal two-thirds of the ileum, extending from Meckel’s diverticulum to 1 cm proximal to the ileocecal junction. The digesta samples were gently flushed with distilled water, pooled within each cage, and immediately frozen at −20 °C until further analysis.

### 2.4. Chemical Analysis and Calculation

A mill grinder (CT293 Cyclotec, Foss Ltd., Hillerød, Denmark) was used to grind the experimental diets before being subjected to nutrient analysis. The ileal digesta samples were freeze-dried to prevent AA denaturation and manually pulverized with a mortar and pestle. The concentrations of dry matter (DM), crude protein (CP), ether extract (EE), ash, neutral detergent fiber (NDF), and acid detergent fiber (ADF) in feed ingredients and experimental diets were analyzed according to AOAC [12] methods 930.15, 990.03, 920.39, 942.05, 2002.04, and 973.18, respectively. The ileal digesta and experimental diets were analyzed for AA concentration using a high-speed amino acid analyzer (LA8080, Hitachi High-Tech Corp., Tokyo, Japan) according to method 982.30 E (a and b) from AOAC [12]. Gross energy was determined using a bomb calorimeter (Parr 6300, Parr Instruments Co., Moline, IL, USA). Chromium was analyzed as described by Fenton and Fenton [13].

The coefficients of the AID and SID of AA in test ingredients and the BEL of AA in birds were calculated using the following equations established by Kong and Adeola [10]:

AID = 1 − [(Cr_input_/Cr_output_)) × (AA_output_/AA_input_)] (1)

BEL of AA (g/kg of DMI) = (Cr_input_/Cr_output_) × (AA_output_) (2)

SID = AID + (BEL/AA_input_) (3)

where Cr_input_ (%) is the chromium concentration in the diet; Cr_output_ (%) is the chromium concentration in the ileal digesta; AA_input_ (%) is the AA concentration in the diet; AA_output_ (%) is the AA concentration in the ileal digesta; DMI refers to the dry matter intake.

### 2.5. Statistical Analysis

The data were analyzed using a one-way ANOVA implemented through the MIXED procedure in SAS version 9.4 (SAS Institute Inc., Cary, NC, USA). Differences among treatment means were identified using Tukey’s HSD test. Each cage served as the experimental unit, and statistical significance was set at a threshold of *p* < 0.05.

## 3. Results and Discussion

The nutrient concentrations in the SBM, FSBM, RM, CM, PKM, corn DDGS, and FM are presented in Table 2. The total AA concentrations in the SBM, FSBM, PKM, corn DDGS, and FM were within the range reported in previous studies, while most total AA concentrations in the RM and CM were lower than those in previous studies [14,15,16,17,18,19,20,21,22,23,24]. The lower total AA concentration in the RM and CM may be attributed to various factors such as cultivars, environmental conditions, and processing of the seeds, as described in previous research [25,26,27]. The concentration of CP, NDF, and ADF in feed ingredients was consistent with the values reported by NRC [14]. The contents of CP, NDF, and ADF in the FSBM were numerically greater than those in the SBM. The fermentation process involving fungi and bacteria typically degrades nutrient compounds; thus, the contents of NDF, ADF, and EE were generally reduced after fermentation [28]. However, although the EE concentration in the FSBM was lower than that in the SBM, the NDF and ADF concentrations in the FSBM were greater than those of the SBM in the current study. This may be attributable to the presence of soybean hulls in the FSBM, which are rich in structural fiber components, whereas the SBM used in the present study was dehulled. Although the hull content of the SBM used for fermentation could not be confirmed, the inclusion of soybean hulls in the FSBM may explain the greater NDF and ADF concentrations observed after fermentation, despite the general expectation that fermentation reduces fiber fractions. Additionally, fermentation is known to increase the dietary CP and total AA concentration, which is consistent with the current study [28,29,30].

The AID and SID values for AA in the protein sources are presented in Table 3 and Table 4, respectively. The AID and SID values of AA for the FM were the greatest, followed by the SBM. The AID and SID values for Arg, Leu, Thr, Val, and Pro in the SBM were greater (*p* < 0.05) values than those in the RM, with no significant differences observed for the remaining AA. The AID and SID of AA in the RM were not different from those of the FSMB, except for Met, Val, Cys, and Pro. The AID and SID values of AA in the RM and FSBM were greater than those of corn DDGS, except for Leu and Pro in the RM and Met and Cys in the FSBM, respectively, while the AID and SID of AA in corn DDGS exhibited greater (*p* < 0.05) AID and SID values compared to the values in the CM and PKM, except for Val and Asp. The CM values were not different from or greater (*p* < 0.05) than those of the PKM, with exception of Lys.

The AID and SID of AA in the test ingredients, except for in the FM, were consistent with previously reported values [14,15,16,17,18,19,20,21,22,23]. The fermented SBM showed comparable AID and SID values for AA compared with the SBM, except for Arg, Met, Thr, Cys, Glu, and Gly, in the current study. The fermentation process has been used to improve nutrient utilization of the SBM by reducing anti-nutrients and decomposing macromolecules [29]. However, the current study found that the AID and SID values for AA in the FSBM were similar to or lower than those in the SBM, which is consistent with previous studies [17,18]. The AID and SID values for AA in the FSBM were comparable to yet numerically lower than those reported in previous studies. This may be associated with the inherent limitations of the SBM for further improvement of AA digestibility, as well as the variability of the fermentation process based on the species of bacteria and fungi, fermentation time, and pH [30].

The AID and SID of AA in corn DDGS were lower than those of the FSBM and RM, a finding that is in agreement with the values reported in previous research [15,20,21,22,23]. This reduction may be partly attributed to the higher fiber concentration in corn DDGS than that in the FSBM, as dietary fiber has been reported to negatively affect AA digestibility of feed ingredients in broilers [31,32]. Dietary soluble fiber increases intestinal viscosity, thereby reducing digesta passage rate and limiting enzyme diffusion and substrate breakdown [33]. Additionally, greater fiber levels have been associated with greater ileal endogenous AA losses, thereby increasing excretion of undigested AA [34]. However, the greater AA digestibility observed in the RM compared with the FSBM despite its higher fiber content indicates that dietary fiber alone does not fully explain differences in AA digestibility among protein sources. These inconsistencies indicate that the relationship between dietary fiber and AA digestibility is complex and warrants further investigation. Accordingly, the CM and PKM, which contained the greatest NDF and ADF content, exhibited the lowest AID and SID values for AA. This finding is consistent with previous studies [15,16,35]. Although total dietary fiber and soluble fiber fractions were not directly quantified in the present study, NDF and ADF are closely related to total fiber content, and soluble fiber has been reported to be positively associated with these fiber fractions [36]. Therefore, it can be reasonably assumed that total and soluble fiber contents increased proportionally in ingredients with higher NDF and ADF concentrations. To allow more precise interpretation of fiber-related effects on AA digestibility, future studies should quantify individual fiber fractions when evaluating ileal digestibility. Collectively, the present findings indicate that high-fiber protein sources generally exhibit lower AA digestibility, suggesting that precise digestibility coefficients can be used to define economically optimal inclusion levels of such ingredients in broiler diets.

In the current study, the FM exhibited the greatest SID values for AA, except for Met and Cys, ranging from 97.4% to 98.7%, while the SID values for AA of the FM in previous research ranged from 65.5% to 88.1% [21,24]. This discrepancy in AA digestibility of the FM between the current study and previous studies necessitates further research. These results indicate that nutrient composition of protein sources can vary due to several factors, such as feed ingredients condition and process method, which can lead to variations in the ileal digestibility of AA for broilers. This variability highlights the continuous need for evaluating AA digestibility of protein sources, particularly for novel feed ingredients. Moreover, precisely determined AA digestibility coefficients provide a scientific basis for utilizing diverse protein sources as alternatives to the soybean meal, enabling more accurate nutrient supply and contributing to economically efficient and sustainable broiler production by reducing excess nutrient excretion.

## 4. Conclusions

In conclusion, the AID and SID of AA were the greatest in the FM, followed by the SBM, FSBM, RM, corn DDGS, CM and the PKM. Furthermore, these results underscore the necessity for continuous evaluation of ileal digestibility of AA in various protein sources, particularly for novel feed ingredients.

## Figures and Tables

**Table 1:** Ingredients composition and nutrient content of experimental diets (as-fed basis).

Ingredient, %	Dietary Treatments							
	NFD	SBM	FSBM	RM	CM	PKM	Corn DDGS	FM
Cornstarch	20.95	48.99	55.92	34.86	-	-	19.86	64.10
Sucrose	64.00	-	-	-	-	-	-	-
SBM	-	43.55	-	-	-	-	-	-
FSBM	-	-	36.51	-	-	-	-	-
RM	-	-	-	58.00	-	-	-	-
CM	-	-	-	-	92.64	-	-	-
PKM	-	-	-	-	-	92.48	-	-
Corn DDGS	-	-	-	-	-	-	72.73	-
FM	-	-	-	-	-	-	-	30.00
Limestone	0.28	-	0.04	-	0.31	-	0.45	-
MCP	2.02	1.56	1.63	1.24	1.15	1.62	1.06	-
Soybean oil	4.00	4.00	4.00	4.00	4.00	4.00	4.00	4.00
Cellulose	5.00	-	-	-	-	-	-	-
Potassium carbonate	0.30	-	-	-	-	-	-	-
Sodium bicarbonate	1.20	-	-	-	-	-	-	-
Potassium chloride	0.30	-	-	-	-	-	-	-
Magnesium oxide	0.20	-	-	-	-	-	-	-
Salt	-	0.20	0.20	0.20	0.20	0.20	0.20	0.20
Vitamin premix	0.50	0.50	0.50	0.50	0.50	0.50	0.50	0.50
Mineral premix	0.50	0.50	0.50	0.50	0.50	0.50	0.50	0.50
Choline chloride	0.25	0.20	0.20	0.20	0.20	0.20	0.20	0.20
Chromic oxide	0.50	0.50	0.50	0.50	0.50	0.50	0.50	0.50
Calculated value, %								
AME, MJ/kg	15.3	14.2	14.7	11.6	15.9	6.5	13.4	15.9
CP	-	19.8	20.0	18.9	20.0	14.0	19.8	20.0
Calcium	0.75	0.75	0.75	1.01	0.75	0.83	0.75	1.18
Non-phytate P	0.42	0.42	0.42	0.42	0.42	0.42	0.41	0.55

AME, apparent metabolizable energy; CM, copra meal; Corn DDGS, corn distillers dried grains with solubles; CP, crude protein; FM, fish meal; FSBM, fermented soybean meal; NFD, nitrogen-free diet; MCP, monocalcium phosphate; PKM, palm kernel meal; RM, rapeseed meal; SBM, soybean meal.

**Table 2:** Nutrient composition of test ingredients (as-fed basis).

Item, %	Test Ingredients						
	SBM	FSBM	RM	CM	PKM	Corn DDGS	FM
DM	96.8	89.0	93.7	91.5	94.3	97.3	97.7
CP	45.9	54.8	34.5	21.8	16.3	27.5	66.8
NDF	9.12	17.83	30.84	49.63	58.48	37.1	24.32
ADF	8.22	12.15	17.7	29.02	37.02	13.36	1.13
Ash	3.91	10.68	6.61	4.42	6.64	7.58	20.89
EE	2.75	1.79	3.70	2.38	8.49	8.19	6.32
Indispensable AA							
Arg	3.04	3.48	1.84	1.77	1.64	1.13	3.93
His	1.08	1.27	0.73	0.31	0.26	0.66	1.28
Ile	1.82	2.34	1.03	0.56	0.45	0.8	2.66
Leu	3.2	4.02	1.94	1.15	0.88	2.77	4.87
Lys	2.69	3.22	1.26	0.41	0.5	0.86	5.48
Met	0.6	0.73	0.57	0.28	0.3	0.6	1.53
Phe	2.11	2.61	1.19	0.81	0.58	1.17	2.58
Thr	1.76	2.08	1.28	0.61	0.47	1.06	2.76
Val	1.97	2.46	1.44	0.91	0.7	1.19	3.06
Dispensable AA							
Ala	1.82	2.26	1.2	0.79	0.55	1.72	4.1
Asp	4.87	5.89	2.04	1.46	1.13	1.61	6.28
Cys	0.68	0.87	0.88	0.34	0.23	0.54	0.77
Glu	7.7	9.14	5.42	3.42	2.57	4.31	9.07
Gly	1.75	2.26	1.45	0.79	0.63	0.95	3.95
Pro	2.2	2.6	1.93	0.75	0.53	2.26	2.43
Ser	2.22	2.71	1.31	0.8	0.61	1.27	2.79
Tyr	1.53	1.86	0.79	0.37	0.27	0.92	2.04

AA, amino acid; ADF, acid detergent fiber; CM, copra meal; Corn DDGS, corn distillers dried grains with solubles; CP, crude protein; DM, dry matter; EE, ether extract; FM, fish meal; FSBM, fermented soybean meal; NDF, neutral detergent fiber; PKM, palm kernel meal; RM, rapeseed meal; SBM, soybean meal.

**Table 3:** Apparent ileal digestibility (%) of amino acids in test ingredients.

Item	Apparent Ileal Digestibility								
	SBM	FSBM	RM	CM	PKM	Corn DDGS	FM ^1^	SEM	*p*-Value
n ^2^	8	8	8	8	8	8	7		
Indispensable AA, %									
Arg	89.4 ^a^	77.9 ^b^	77.4 ^b^	70.5 ^b^	71.5 ^b^	70.7 ^b^	98.5 ^a^	2.44	<0.0001
His	80.7 ^b^	80.8 ^b^	80.6 ^b^	54.0 ^c^	40.7 ^d^	78.6 ^b^	98.2 ^a^	3.10	<0.0001
Ile	83.4 ^b^	81.7 ^b^	81.0 ^b^	66.0 ^c^	56.8 ^c^	62.9 ^c^	98.1 ^a^	2.51	<0.0001
Leu	83.1 ^b^	81.6 ^bc^	72.7 ^cd^	57.7 ^e^	67.6 ^de^	83.6 ^b^	98.3 ^a^	2.55	<0.0001
Lys	89.2 ^ab^	83.3 ^bc^	80.8 ^bcd^	57.4 ^e^	72.2 ^d^	78.5 ^cd^	98.7 ^a^	2.49	<0.0001
Met	88.0 ^a^	76.5 ^c^	84.3 ^ab^	78.4 ^bc^	82.1 ^abc^	85.4 ^a^	87.9 ^a^	1.61	<0.0001
Phe	81.2 ^b^	82.3 ^b^	77.9 ^bc^	66.3 ^d^	71.2 ^cd^	67.7 ^d^	98.0 ^a^	2.31	<0.0001
Thr	85.6 ^ab^	72.8 ^c^	73.8 ^c^	64.3 ^cd^	57.3 ^d^	75.3 ^bc^	97.4 ^a^	2.87	<0.0001
Val	80.6 ^b^	79.4 ^bc^	70.3 ^d^	80.3 ^b^	74.7 ^bcd^	71.5 ^cd^	98.0 ^a^	2.03	<0.0001
Dispensable AA, %									
Ala	81.3 ^b^	77.4 ^b^	71.1 ^bc^	62.2 ^cd^	55.8 ^d^	78.9 ^b^	98.0 ^a^	2.81	<0.0001
Asp	83.3 ^b^	81.7 ^b^	78.8 ^b^	74.6 ^bc^	66.4 ^cd^	59.8 ^d^	97.7 ^a^	2.21	<0.0001
Cys	75.6 ^a^	54.4 ^b^	75.5 ^a^	53.5 ^b^	50.9 ^b^	72.5 ^a^	77.1 ^a^	3.29	<0.0001
Glu	88.2 ^ab^	74.4 ^c^	79.6 ^bc^	74.4 ^c^	77.0 ^c^	80.2 ^bc^	98.3 ^a^	2.51	<0.0001
Gly	88.3 ^ab^	74.2 ^c^	79.7 ^bc^	72.4 ^c^	70.6 ^c^	77.2 ^c^	97.8 ^a^	2.47	<0.0001
Pro	85.0 ^b^	83.3 ^b^	69.4 ^d^	74.1 ^cd^	66.5 ^d^	78.4 ^bc^	98.0 ^a^	2.12	<0.0001
Ser	78.6 ^b^	77.8 ^b^	75.2 ^bc^	70.1 ^bcd^	65.5 ^cd^	63.8 ^d^	97.6 ^a^	2.44	<0.0001
Tyr	82.2 ^b^	83.1 ^b^	78.4 ^bc^	67.7 ^d^	56.3 ^e^	71.7 ^cd^	98.5 ^a^	2.44	<0.0001

AA, amino acid; CM, copra meal; Corn DDGS, corn distillers dried grains with solubles; FM, fish meal; FSBM, fermented soybean meal; PKM, palm kernel meal; RM, rapeseed meal; SBM, soybean meal; SEM, standard error of the mean. ^1^ The standard error of the mean (SEM) for the FM group was calculated separately due to the difference in the number of observations. The respective SEM values for the amino acids were as follows: Arg (2.60), His (3.29), Ile (2.67), Leu (2.72), Lys (2.65), Met (1.72), Phe (2.47), Thr (3.07), Val (2.17), Ala (2.98), Asp (2.36), Cys (3.47), Glu (2.68), Gly (2.63), Pro (2.26), Ser (2.61), and Tyr (2.60). ^2^ Number of observations. ^a–e^ Least squares means within a row without a common superscript differ (*p* < 0.05).

**Table 4:** Standardized ileal digestibility (%) of amino acids in test ingredients.

Item	Standardized Ileal Digestibility								
	SBM	FSBM	RM	CM	PKM	Corn DDGS	FM ^1^	SEM	*p*-Value
n ^2^	8	8	8	8	8	8	7		
Indispensable AA, %									
Arg	89.4 ^a^	78.0 ^b^	77.4 ^b^	70.5 ^b^	71.5 ^b^	70.7 ^b^	98.6 ^a^	2.44	<0.0001
His	80.8 ^b^	80.9 ^b^	80.7 ^b^	54.2 ^c^	40.9 ^d^	78.6 ^b^	98.3 ^a^	3.09	<0.0001
Ile	83.5 ^b^	81.7 ^b^	81.1 ^b^	66.1 ^c^	56.9 ^c^	63.0 ^c^	98.2 ^a^	2.50	<0.0001
Leu	83.2 ^b^	81.7 ^bc^	72.8 ^cd^	57.8 ^e^	67.7 ^de^	83.6 ^b^	98.3 ^a^	2.54	<0.0001
Lys	89.2 ^ab^	83.3 ^bc^	80.8 ^bcd^	57.5 ^e^	72.2 ^d^	78.6 ^cd^	98.7 ^a^	2.49	<0.0001
Met	88.0 ^a^	76.5 ^c^	84.3 ^ab^	78.5 ^bc^	82.1 ^abc^	85.4 ^a^	87.9 ^a^	1.60	<0.0001
Phe	81.2 ^b^	82.4 ^b^	78.0 ^bc^	66.4 ^d^	71.3 ^cd^	67.8 ^d^	98.0 ^a^	2.31	<0.0001
Thr	85.7 ^ab^	72.8 ^c^	73.9 ^c^	64.4 ^cd^	57.5 ^d^	75.4 ^bc^	97.5 ^a^	2.87	<0.0001
Val	80.6 ^b^	79.4 ^bc^	70.4 ^d^	80.3 ^b^	74.8 ^bcd^	71.5 ^cd^	98.1 ^a^	2.03	<0.0001
Dispensable AA, %									
Ala	81.3 ^b^	77.4 ^b^	71.1 ^bc^	62.3 ^cd^	55.9 ^d^	78.9 ^b^	98.0 ^a^	2.80	<0.0001
Asp	83.4 ^b^	81.8 ^b^	78.9 ^b^	74.6 ^bc^	66.5 ^cd^	59.9 ^d^	97.7 ^a^	2.20	<0.0001
Cys	75.7 ^a^	54.5 ^b^	75.6 ^a^	53.6 ^b^	51.1 ^b^	72.6 ^a^	77.2 ^a^	3.28	<0.0001
Glu	88.2 ^ab^	74.5 ^c^	79.7 ^bc^	74.4 ^c^	77.1 ^c^	80.2 ^bc^	98.3 ^a^	2.51	<0.0001
Gly	88.4 ^ab^	74.3 ^c^	79.8 ^bc^	72.4 ^c^	70.6 ^c^	77.3 ^c^	97.9 ^a^	2.47	<0.0001
Pro	85.0 ^b^	83.3 ^b^	69.5 ^d^	74.2 ^cd^	66.6 ^d^	78.4 ^bc^	98.1 ^a^	2.12	<0.0001
Ser	78.6 ^b^	77.9 ^b^	75.2 ^bc^	70.2 ^bcd^	65.6 ^cd^	63.9 ^d^	97.6 ^a^	2.44	<0.0001
Tyr	82.3 ^b^	83.2 ^b^	78.5 ^bc^	67.8 ^d^	56.5 ^e^	71.7 ^cd^	98.6 ^a^	2.43	<0.0001

AA, amino acid; CM, copra meal; Corn DDGS, corn distillers dried grains with solubles; FM, fish meal; FSBM, fermented soybean meal; PKM, palm kernel meal; RM, rapeseed meal; SBM, soybean meal; SEM, standard error of the mean. ^1^ The standard error of the mean (SEM) for the FM group was calculated separately due to the difference in number of observations. The respective SEM values for the amino acids were as follows: Arg (2.60), His (3.28), Ile (2.66), Leu (2.71), Lys (2.65), Met (1.72), Phe (2.47), Thr (3.06), Val (2.17), Ala (2.97), Asp (2.36), Cys (3.46), Glu (2.68), Gly (2.63), Pro (2.26), Ser (2.60), and Tyr (2.59). ^2^ Number of observations. ^a–e^ Least squares means within a column without a common superscript differ (*p* < 0.05).

## Data Availability

The data presented in this study are available upon request from the corresponding author.

## Abbreviations

The following abbreviations are used in this manuscript:

AA	Amino acids
ADF	Acid detergent fiber
AID	Apparent ileal digestibility
BEL	Basal endogenous losses
CM	Copra meal
CP	Crude protein
DDGS	Distillers dried grains with solubles
DM	Dry matter
EE	Ether extract
FM	Fish meal
FSBM	Fermented soybean meal
NDF	Neutral detergent fiber
PKM	Palm kernel meal
RM	Rapeseed meal
SBM	Soybean meal
SID	Standardized ileal digestibility
